# Research progress on the tsRNA classification, function, and application in gynecological malignant tumors

**DOI:** 10.1038/s41420-021-00789-2

**Published:** 2021-12-14

**Authors:** Jing-tao Wen, Zheng-hao Huang, Qian-hui Li, Xi Chen, Hong-lei Qin, Yang Zhao

**Affiliations:** Department of Obstetrics and Gynecology, Department of Gynecologic Oncology Research Office, Key Laboratory for Major Obstetric Diseases of Guangdong Province, The Third Affiliated Hospital of Guangzhou Medical University, Guangzhou, 510150 China

**Keywords:** Diagnostic markers, Ovarian cancer

## Abstract

A large number of small non-coding RNAs derived from tRNAs, called tRNA-derived small RNA (tsRNAs), have been identified by high-throughput RNA sequencing of cell lines. Further research has revealed that they are not produced via random tRNA degradation, but through degradation by specific nuclease cleavages, such as Elac Ribonuclease Z 2 (ELAC2)/RNase Z, RNase L, Dicer, and angiogenin (ANG), the tsRNAs can be classified into the following types based on the location from which they have been derived from the parental tRNA: tRF-1s, tRF-3s, tRF-5s, tiRNA, and tRF-2s/i-tRFs. Moreover, tsRNAs are a type of small RNAs with diverse functions, including gene expression regulation, anti-apoptosis, translation inhibition, participation in epigenetic regulation, initial virus reverse transcription, promote virus replication and cell-to-cell communication. Certain types of tsRNAs are overexpressed in cancer tissues, but are underexpressed in normal tissues. Therefore, the relationship between tsRNAs and the occurrence and development of cancer has attracted significant research attention. Research advancements have contributed to further discoveries of the biological activities of tsRNAs, but the mechanisms of their biogenesis and functions have not been fully elucidated. This article reviews the classification and biological functions of tsRNAs, and introduces the research progress in gynecological malignancies.

## Facts

- tsRNAs are not random degradation fragments from tRNAs;
- tsRNAs can be classified into tRF-1s, tRF-3s, tRF-5s, tiRNA, and tRF-2s based on the location from which they have been derived from the parental;
- tsRNAs participate in gene expression regulation, anti-apoptosis, translation inhibition, and virus infection;
- tsRNAs are involved in the occurrence and development of cancer.

## Open Questions

- What mechanism is responsible for the production of each type of tsRNA?
- How does tsRNA participate in gene regulation, anti-apoptosis, translation inhibition, and virus infection?
- What is the application prospect of tsRNA as a diagnostic marker and target for the treatment of gynecological malignancies?

## Introduction

RNA sequencing (RNA-seq) is widely used in molecular biology for research on the functions of RNA at all levels. This approach reveals the diversity of mRNA splicing and the mechanisms by which ncRNA and enhancer RNA regulate gene expression.

**Fig. 1:**
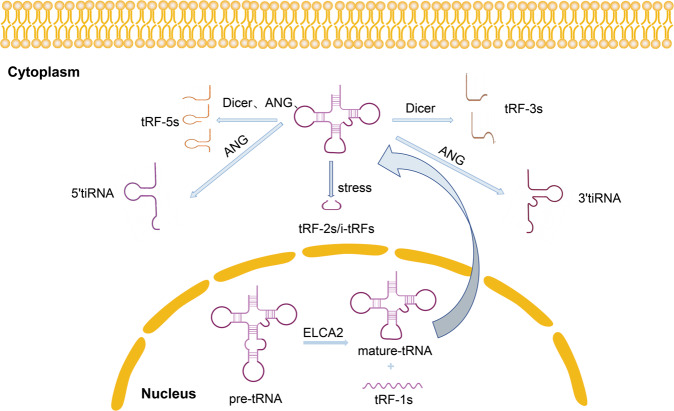
Source and structure of different types of tsRNA. tRF-1s is derived from pre-tRNA by ELCA2- mediated cleavage in the nucleus. tRF-3s is derived from the 3′-end of mature tRNA and can be divided into two types. tRF-5s can be produced by Dicer and ANG cleavage, starting from the 5′-end of the mature tRNA, including the complete structure of the 5′-end, and terminating before the anticodon loop tRF-5s can be divided into three sub-types: tRF-5a, tRF-5b, and tRF-5c. The tiRNA is derived from mature tRNA by ANG-mediated cleavage in the cytoplasm and can be divided into 5’tiRNA and 3’tiRNA. tRF-2s/i-tRFs are generated from the anticodon loop of mature tRNA.

The advancements in the RNA-seq technique have contributed to the identification of small non-coding RNAs with a length of approximately 13–48 nts. Further sequencing analysis suggested that the abundance of this type of RNA is second only to miRNAs and is derived from the precise processing of the 5′- or 3′-ends of mature tRNA or pre-tRNA, producing several types of tRNA-derived small RNA (tsRNAs). One subtype is termed tRNA-derived RNA fragments (tRFs) includes tRF-1s, tRF-3s, and tRF-5s [1]. Meanwhile, other tsRNA types have been found, such as tRF-2s or i-tRFs, stress-induced tRNA (tiRNA) (Fig. 1). The evolution of tsRNAs from bacteria to humans is highly conserved [2]. tsRNAs have diverse functions, such as gene expression, translation inhibition, and virus infection [3, 4]. An earlier study showed that tRNA cleavage is one of the mechanisms of tsRNA biogenesis [5]. Studies have shown that the generation of some tsRNAs, such as tRF-3s and tRF-5s, is related to multiple RNase family members, including ANG and Dicer, but the role of Dicer in tsRNA generation may be a particular case rather than a conventional one because the presence of tsRNA is also easily detected in Dicer knockout (Dicer−/−) mouse fibroblasts, it indicates that the production of this type of tsRNA is independent of Dicer processing [2, 6–8]. tsRNAs are derived from most tRNA genes in unequal abundance, different sizes, and various regions. Their biogenesis is regulated in many ways and has well-defined 5′- and 3′-ends; hence, tsRNAs are not a product of random cleavage of tRNAs [9]. In view of the diversity of the types and functions of tsRNAs, which are increasingly being valued by researchers, numerous tsRNAs have been proven to play an important role in the occurrence and development of malignant tumors (Table 1). This article reviews the structure, source, function of various tsRNAs, and introduces the research progress in common gynecological malignancies.

**Table 1:** The role of tsRNA in cancer.

tsRNA name	tsRNA type	Parental tRNA	Cancer	Function	Reference
tRF1001	tRF-1s	tRNA-Ser-TGA	Prostate cancer	Promotes cell proliferation	[1]
tRF3007	tRF-3s	tRNA-Gly-TCC	Breast cancer	Inhibit cell migration and invasion	[78]
tRF^Glu^	5’tiRNA	tRNA-Glu-YTC	Breast cancer	Inhibit cell invasion	[15]
tRF-Leu-CAG	5’tiRNA	tRNA‐Leu‐CAG	Lung cancer	Promotes cell proliferation and cell cycle	[79]
5′-tRF-GlyGCC	tRF-5s	tRNA-Gly-GCC	Colorectal cancer	Carcinogenesis	[75]
5′-tRF-GlyGCC	tRF-5s	tRNA-Gly-GCC	Cervical cancer	Promotes tumor progression	[76]
tRF3017a	tRF-3s	tRNA-Val-TAC	Gastric cancer	Promotes metastasis	[80]
tRF3019	tRF-3s	tRNA-Pro-/	Adult T-cell leukemia/lymphoma	Carcinogenesis	[65]
5′tRNA4-Val-AAC	5’tiRNA	tRNA-Val-AAC	Clear cell renal cell carcinoma	Suppresses tumor progression	[81]
LeuCAG3′tsRNA	tRF-3s	tRNA-Leu-CAG	Hepatocellular carcinoma	Promotes tumor growth	[82]
ts3	unknow	tRNA-Val-/	Ovarian cancer	Promotes tumor progression	[74]
CU1276	tRF-3s	tRNA-Gly-GCC	B cell lymphoma	Suppresses cell proliferation	[83]

## Source and structure of tsRNA

### Source and structure of tRF-1s

tRF-1s is derived from pre-tRNA, and its 3′-end contains an RNA polymerase III transcription-termination sequence. The first tRF-1 discovered was named tRF-1001. Its sequence started from the 3′-end of the mature tRNA exactly before the CCA sequence was added. There are 5–6 consecutive thymines at the 3′-end of the corresponding locus, which is the typical termination signal for RNA polymerase III transcription [10]. These sequence characteristics indicate that tRF-1s are derived from the 3′-tail sequence of pre-tRNA, which is released by the precise cleavage of the tRNA endonuclease ELAC2 during the maturation of the 3′-end of the tRNA. The reduction of tRF-1001 and the concomitant accumulation of pre-tRNA also confirmed this notion in an experiment with ELAC2 knockdown [1]. ELAC2 is considered a susceptibility gene for prostate cancer [11]. Later, the protein encoded by this gene was discovered to have a C-terminal domain with tRNA 3′-end-processing endoribonuclease activity, which catalyzes the removal of the 3′-tail sequence sequence from pre-tRNA. The production of tRF-1s is low since only 14.6% of tRNA loci can produce tRF-1. These differences show that the molecules of tRF-1s are selectively processed or retained in cells to achieve specific biological functions [1].

In tRFdb, according to their order of discovery, tRF-1s have been consecutively named tRF-1001, tRF1003, tRF1004, etc. [12]. So far, 32 human tRF-1s have been identified in tRFdb. The number of tRF-1s may differ in other databases, such as MINTbase [13].

The length of tRF-1s varies from 14 to 30 nucleotides, and its 3′-end contains at least three consecutive uracils. Among the 622 loci in the human genome, at the 3′-end, the distance between the tRNA and the RNA polymerase III termination signal is variable. The sequence of each tRF-1 is the same as the tail sequence of the corresponding tRNA, which is a sequence feature of tRF-1s.

### Source and structure of tRF-3s

tRF-3s is derived from the 3′-end of mature tRNA and can be divided into two types based on its cutting position (U/A or U/U): tRF-3a and tRF-3b with lengths of 18 and 22 nucleotides, respectively. tRF-3s can be produced by Dicer cutting of the TΨC loop of mature tRNA [13]. The 22- nucleotide tRF-3b is produced by cleavage between the 54th and 55th nucleotides on the TΨC loop. The 18- nucleotide tRF-3a is generated by cleavage between the 58th and 59th nucleotides on the TΨC loop. The ends of the sequence contain the CCA sequence, added during mature tRNA processing. The number of tRF-3s is significantly higher than that of tRF-1s, and 461 tRF-3s have been currently identified and recorded in tRFdb.

### Source and structure of tRF-5s

tRF-5s can be produced by Dicer and ANG cleavage, starting from the 5′-end of the mature tRNA, including the complete structure of the 5′-end, and terminating before the anticodon loop [8]. Based on its different termination sites, tRF-5s can be divided into three sub-types: tRF-5a, tRF-5b, and tRF-5c, with lengths 14–16, 22–24, and 28–30 nucleotides, respectively [3]. The number of tRF-5s is also significantly higher than that of tRF-1s and tRF-3s, reaching 539 species.

### Source and structure of tiRNA

Under hypoxic nutrient deficiency or other stress conditions, tRNA can be cleaved in the anticodon loop to form tiRNA by a specific nuclease that recognizes specific tRNA structural sites [14, 15]. The tiRNA is also called tRNA halves in other literature and can be divided into two subtypes: 5’tiRNA and 3’tiRNA. Under stress conditions, the specific nuclease that induces tRNA cleavage and produces tiRNA is angiogenin (ANG). The evidence comes from two aspects [3]: first, 3’tiRNA has a 5′hydroxyl label, while 5’tiRNA has a cyclic phosphate, which is the typical expression of cleavage by the RNase A nuclease family; second, the knockdown of angiogenin considerably reduces the level of tiRNA. Stress conditions that promote tRNA’s angiogenin (ANG) cleavage to produce tiRNAs include hypoxia, starvation, viral infection, arsenic exposure, heat shock, and heavy metal toxicity [16**–**21].

Angiogenin (ANG) has ribonuclease activity and can thus be transferred to the cytoplasm and accumulate in stress granules (SG) under stress conditions [22]. The production of tiRNA is not a product of random degradation of RNA. So, by what mechanism does ANG control its nuclease activity? Ribonuclease inhibitor 1 (RNH1) is a protein widely expressed in various tissues that can bind to and inhibit pancreatic ribonuclease [23]. Immunoprecipitation under normal physiological conditions has confirmed that RNH1 interacts with cytoplasmic ANG but is not related to nuclear ANG. Therefore, nuclear ANG has enzymatic activity, but cytosolic ANG is inhibited. In contrast, RNH1 interacts with nuclear ANG under stress conditions but is not related to cytoplasmic ANG. Therefore, nuclear ANG is suppressed, but cytoplasmic ANG is not. RNH1 knockout would change the cellular localization of ANG and terminate the biological activity of ANG. Therefore, the cellular activity of ANG is suggested to be controlled by its localization and association with RNH1 [24]. In addition, a number of animal experiments have revealed that the methylation modification of mature tRNA by DNA methyltransferase 2 (DNMT2) or cytosine-5 methyltransferase NOP2/Sun RNA Methyltransferase 2 (NSUN2) promotes the stability of mature tRNA and provides resistance to the cleavage of ANG [25**–**27]. However, evidence has shown that certain tiRNAs do not depend on ANG cleavage; the sequence of such tiRNAs is longer than that of conventional tiRNAs, suggesting that the corresponding nuclease cleavage site should be outside of the anticodon loop [28, 29]. In the case of ANG knockout, only part of the expression level of tsRNA changes, indicating that ANG is only responsible for producing part of tsRNA rather than all tsRNAs, and there should be other endonucleases involved in the production of tsRNA [30]. Studies have shown that RNase L is activated explicitly during dsRNA reaction, which can cleave tRNA at specific sites of the anticodon loop to produce tiRNA [31].

The 5′-tiRNA sequence is shorter than that of 3’tiRNA, approximately 31–36 nt versus 36–41 nt, correspondingly. Due to the cleavage position of ANG, the 5′-end of 5’-tiRNA is a single 5′-monophosphate, and the 3′-end may be a 2′, 3′-cyclic phosphate. The 5′-end of 3′-tiRNA is a hydroxyl group. The difference in the sequence indicates that the two types of tiRNA have diverse functions [21].

Sex hormone-dependent tRNA-derived RNAs (SHOT-RNAs) is a specific tiRNA type, produced by ANG cleavage of fully aminoacylated mature tRNA, is abundantly expressed in hormone-dependent breast and prostate cancer [32]. The mRNA expression levels of ANG and RNH1 in LNCaP-FGC cells did not change in hormone-free medium culture, indicating that ANG and RNH1 do not directly regulate the expression of SHOT-tiRNA.

### Source and structure of tRF-2s/i-tRFs

tRF-2s/i-tRFs are a class of atypical tsRNA derived from tRNAs. In other literature, they are also divided into different types of tsRNAs. Their structural characteristics are that they contain the anticodon loop and stem of tRNA but do not extend to the 5′-end and 3′ -ends. Its production may be related to hypoxia stress stimulation, but the specific mechanism remains unclear. The i-tRFs are longer than tRF-2s, and the cleavage position is located in D-loop and T-loop. It has been suggested that the length of this class of tsRNA is also closely related to some disease states [3, 15, 33–35].

## tsRNA functions

### Gene expression regulation

**Fig. 2:**
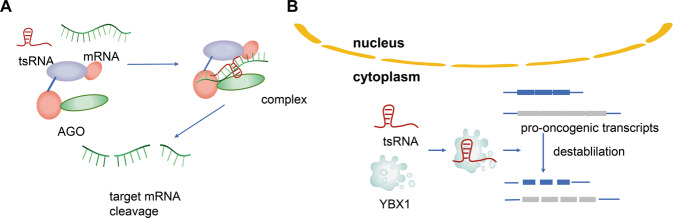
The function of tsRNA on gene expression regulation. **A** tsRNA can be loaded into AGO family proteins in a miRNA-like manner to regulate the expression of target genes. **B** tsRNA binding to YBX-1, change the stability of oncogene mRNA, affect the expression of the oncogene.

The tsRNA is an abundantly expressed non-coding small RNA that can bind to mRNA in a miRNA-like manner to regulate target gene expression. It is noteworthy that tsRNAs can be loaded into AGO family proteins (Fig. 2A), and the combination of the two regulates gene expression through trans-silencing [36]. An analysis of Photoactivatable 164 Ribonucleoside-Enhanced Crosslinking and Immunoprecipitation (PAR-CILP) data showed that tRF-5s and tRF-3s contain abundant seed sequences that match the central region of RNA cross-linking, indicating that tRF-5s and tRF-3s are associated with AGO1, AGO3, and AGO4, but not with AGO2; thousands of tsRNA-mRNA chimeras were found in the Crosslinking, Ligation, and Sequencing of Hybrids (CLASH) data [2]. This strong evidence shows that tsRNAs target and regulate RNA functions in a miRNA-like manner, thereby regulating gene expression. In addition, a previous investigation found that a group of specific tsRNAs containing the CU-box motif binds to the cancer-promoting RNA-binding protein Y-box Binding Protein 1(YBX-1), replacing YBX-1 transcripts from the YBX-1 complex [15]. At the post-transcriptional level this tsRNAs group inhibited the expression of the YBX-1 transcript, changed the stability of oncogene mRNA, affected the expression of oncogene, and finally induced changes in the phenotype of the cancer cells (Fig. 2B).

### Protein translation inhibition

**Fig. 3:**
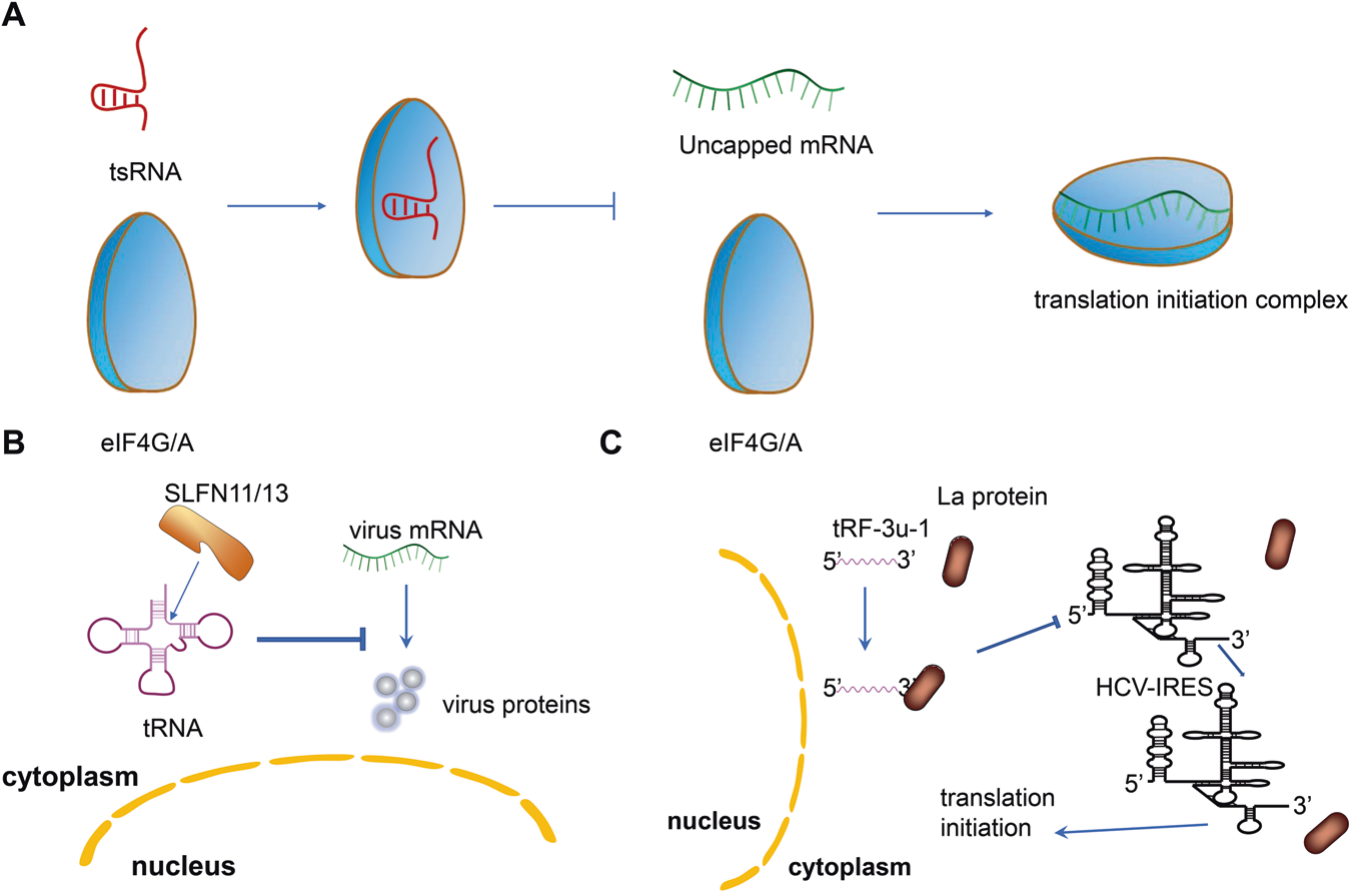
The function of tsRNA on protein translation inhibition. **A** tsRNA binds to eIF4G/A complex displaces mRNA without cap structure to cause translational inhibition. **B** SLFN11/13 inhibits virus proteins synthesis by cleaving tRNA. **C** tRF-3U-1 inhibits HCV IRES-mediated translation by isolating limited La/SSB in the cytoplasm.

Under stress conditions, protein translation is reprogrammed to facilitate the survival of mammalian cells [37]. Stress conditions can promote the production of tsRNAs (mainly tiRNA), and tiRNA plays an important role in reprogramming protein translation through a pathway independent of phosphorylated eIF2α [38]. In a pull-down experiment in which biotin-labeled RNA was used, tiRNA quantitatively displaced Eukaryotic Translation Initiation Factor 4 G/A (eIF4G/A) from the mRNA without cap-structure, but only a small part of eIF4G was displaced from mRNA with a cap-structure. The displacement of eIF4G/A leads to a decrease in the degree of eIF4E displacement, which reduces the affinity of eIF4E to interact with the cap. This outcome suggests that tiRNA indirectly or directly binds to eIF4G, eIF4A, or the eIF4G/A complex, causing translational inhibition (Fig. 3A). tRNA contains many highly conserved residues which are necessary for their correct three-dimensional folding and translation recognition mechanism [39]. In addition, 5′-tiRNA assembles a unique G-quadruplex structure. RNA G-quadruplex (RG4) is a critical component of the cellular stress response, which is necessary for tsRNAs function in the regulation of mRNA translation [40]. The source of tRF-5s is similar to that of 5′tiRNA. It is speculated that tRF-5s also has the function of inhibiting protein translation. Studies have shown that most tRF-5s can indeed inhibit protein translation, which is caused by a conservative sequence or structural features (“GG” dinucleotides) conferred [41]. Previous research already confirmed the protein that binds to mammalian tsRNAs to elucidate the underlying mechanism driving translational inhibition [42].

The mechanism of tsRNA inhibiting protein translation may also apply to the tRNA fragments cleaving by Schlafen III (SLNF III) family proteins SLFN13, and its C-terminal region is homologous to the RNA helicase family I. Studies have proved that SLFN13 is a new tRNA targeted ribonuclease to inhibit the replication and activity of the virus by cleaving tRNA, leading to the inhibition of overall translation and has potent anti-HIV activity [43, 44]. It also found that the position of SLFN13 cleaving tRNA is next to the T loop of tRNA. SLFN 11 also belongs to the SLNF III protein family, which can selectively inhibit HIV protein synthesis by binding to tRNA in a codon usage-dependent manner [45]. Moreover, its homology with SLFN 13 is very high, up to 85%, so we have reason to believe that SLNF 11 may have similar functions to SLFN13. Further studies are needed to clarify the exact antiviral mechanism of SLFN11 and SLFN 13 (Fig. 3B).

Studies have proved that tRF_U3_1 has a particular interaction with La/SSB through various experimental methods to verify the interaction [4, 46, 47]. As a molecular chaperone, La/SSB could bind to the stem-loop IV of HCV internal ribosome entry site (IRES) to promote IRES-mediated translation initiation [48]. tRF_U3_1 could show a similar inhibitory effect on poliovirus IRES, and tRF-3U-1 is stable due to the presence of La/SSB in the cytoplasm. tRF-3U-1 inhibits HCV IRES-mediated translation by isolating limited La/SSB in the cytoplasm. Therefore, tRF_U3_1 could play a significant negative regulatory role in La/SSB-dependent virus proteins translation (Fig. 3C).

### Cell apoptosis inhibition

**Fig. 4:**
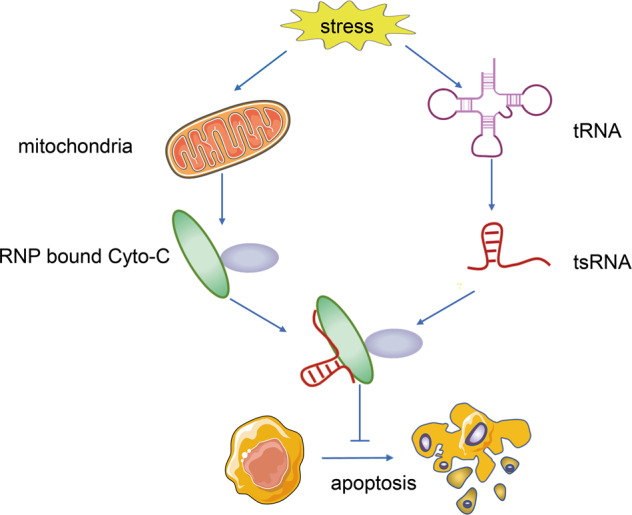
The function of tsRNA on Cell apoptosis inhibition. Under stress condition, tsRNA bind to RNP with cytochrome C to form a complex result in inhibiting cell apoptosis.

Studies have shown that the combination of tRNA and cytochrome C weakens the activities of the combination of cytochrome C and Apaf-1, thereby blocking the formation of apoptotic bodies and the activation of caspase-9, and resulting in cell survival promotion [49]. Whether tsRNA produced by tRNA cleavage under stress conditions retains the anti-apoptotic function of full-length tRNA has attracted appreciable research interest. Further investigations revealed that the accumulation of angiogenin-induced tiRNA is accompanied by an increased survival rate of hyperosmotic stress mouse embryonic fibroblasts. The RNP complex of small RNA and cytochrome C is found in the cells treated with angiogenin under stress conditions. The analysis of small RNA sequencing results of the RNA obtained by immunoprecipitation of the complex showed that the small RNA included tiRNA produced by angiogenesis and cleavage of tRNA. Therefore, a mechanism is believed to exist associated with the binding of cytochrome C and tiRNA inhibition of endogenous apoptosis [50] (Fig. 4).

In addition, tiRNA can selectively enhance the translation of mRNAs involved in anti-apoptotic genes, so that cells can survive under conditions that are not conducive to survival [51]. Generally, the translation of eukaryotic mRNA requires a 5′-cap to mediate ribosome binding, but exceptions are present in eukaryotes and viruses. For example, some genes have a short RNA sequence (approximately 150–250 bp) at the 5′-end. This RNA sequence type can fold into a structure similar to that of the initial tRNA, thereby mediating ribosomal RNA binding and initiating protein translation. This untranslated RNA is called the internal ribosome entry site (IRES). Therefore, in the case of 5′-cap-dependent translation downregulation during cellular stress, IRES can be used for the translation of key regulatory proteins [52]. The genes translated by IRES are usually related to cell survival and apoptosis. This type of genes includes Bcl-2, BAG-1, HiaP2, XIAP, HIF-1a., VEGF, c-Myc, c-Myb, etc [53**–**60].

### Participation in epigenetic regulation

**Fig. 5:**
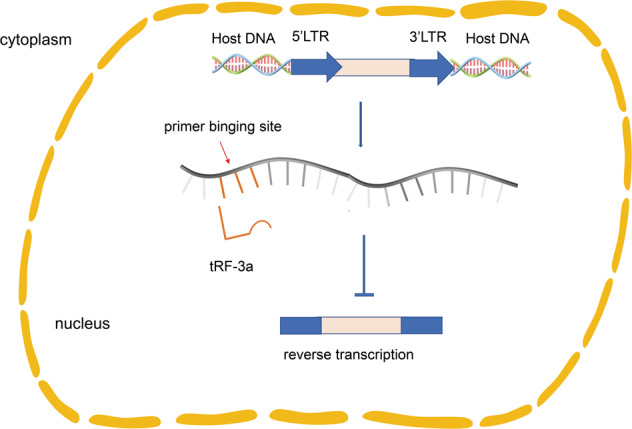
The function of tsRNA on participation in epigenetic regulation. tRF-3a inhibits long terminal repeat (LTR)-retrotransposon or endogenous retrovirus (ERV) activity by targeting the highly conserved primer binding site of LTR-retrotransposon.

The genetic significance of transposons is related to their involvement in insertion mutations, new gene formation, chromosomal aberrations, and biological evolution. They have a substantial impact on the epigenetic control of the genome. The body strictly controls its activities: the transcription of transposons is subject to inhibition of genetic markers such as histone modification and DNA methylation [61]. Studies have shown that other mechanisms exist for transposon activity regulation. To initiate proliferation, long terminal repeat retrotransposons (LTR-retrotransposons) are dependent on reverse-transcribed tRNA availability. In the absence of epigenetic transcriptional inhibition, tRF-3a can strongly inhibit mouse LTR-retrotransposon or endogenous retrovirus (ERV) activity by targeting the highly conserved primer binding site of LTR-retrotransposon (Fig. 5). By transposition reporter gene detection analysis, tsRNA was found to act on any LTR-retrotransposon with replication capability. Meanwhile, tRF-3b reduced the level of the RNA and the protein of the coding-capable autonomous original by post-transcriptional silencing, affecting the transposon expression [62].

### Initial virus reverse transcription and promote virus replication

**Fig. 6:**
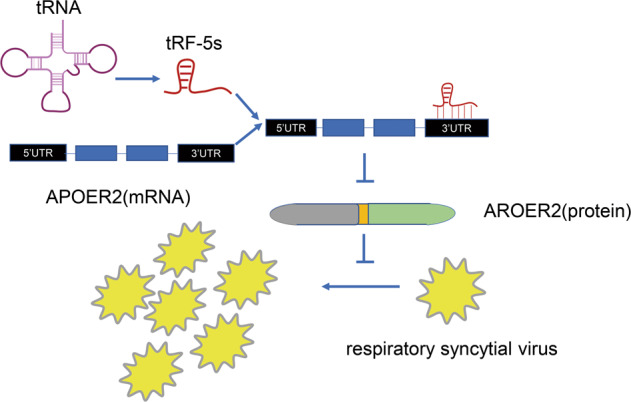
The function of tsRNA on virus infection. tRF-5s recognize the target site in the 3’UTR of apolipoprotein E receptor 2 (APOER2) mRNA. The suppression of AROER2 translation could promote RSV replication.

As mentioned above, tsRNAs produce antiviral effects by inhibiting the protein synthesis of HIV. However, previous works suggest that some tsRNA can also trigger reverse transcription and promote virus replication after virus infection. It was found that the respiratory syncytial virus (RSV) infection can induce cells to produce specific types of tsRNA [63]. Particular tsRNA antisense oligonucleotides or scrambled control oligonucleotides can treat infectious virus particles produced by cells. After apparent tsRNA silence, the copies of RSV were significantly reduced; treatment with specific tsRNAs or negative control mimics showed a significant increase in the copies of RSV. The results strongly proved that a particular tsRNA type could promote RSV replication. Another study showed that the 3′-part of tRF-5Glu-CTC can recognize the target site in the 3′UTR of apolipoprotein E receptor 2 (APOER2) mRNA. As a host anti-RSV protein, APOER2 can promote RSV replication after its suppression [64] (Fig. 6). Ruggero et al. explored microRNA (miRNA) and tsRNA in HTLV-1 infected cells [65]. Their results suggest that the expression of several sequences derived from tRNAs are significantly upregulated in uninfected and infected cells. One of the most abundant tRNA fragments (tRF-3019) comes from the 3’-end of tRNA-proline. The primer binding site of tRF-3019 and HTLV-1 showed perfect sequence complementarity. In vitro reverse transcriptase assay results confirmed that tRF-3019 can initiate reverse transcription of HTLV-1. tRNA-proline and tRF-3019 were detected in virus particles isolated from HTLV-1 infected cells. These findings suggest that tRF-3019 may play an essential role in initiating HTLV-1 reverse transcription.

### Cell-to-cell communication

**Fig. 7:**
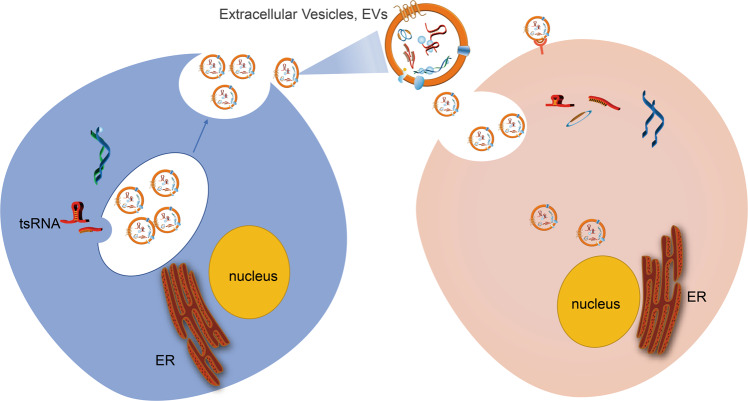
The function of tsRNA on cell-to-cell communication. tsRNA exists stably in extracellular vesicles and plays an essential role in intercellular communication.

RNAs can exist in microvesicles and exosomes, which are called extracellular RNA (exRNA), and can be found in almost all body fluids [66]. It is exciting that this exRNA can affect cell behavior as a signal molecule and plays an essential role in intercellular communication [67, 68]. Because exRNAs can exist stably in their transport carrier extracellular vesicles (EVs), it has the potential to become a diagnostic biomarker of various diseases [69]. Studies have shown that tsRNAs are more enriched than other types of RNA in the EVs produced by cell lines or peripheral blood cells [69, 70]. (Fig. 7). Further studies showed that immune activation signal stimulated T cells could enhance the secretion of extracellular vesicles containing tsRNAs, resulting in the inhibition of T cell activation and cytokine production [69]. Another study showed that 5’tiRNA-Gly from tRNA-Gly was secreted in a concentration-dependent manner in EVs. The production rate of 5’tiRNA-Gly in stressed cells was high, with good thermodynamics and enzyme stability. It was pointed out that the expression of 5’tiRNA-Gly was upregulated during acute stress and could mediate intercellular communication [71].

## Current research status of tsRNA in gynecological malignancies

In 2020, the number of new cancers in women worldwide reached 9.23 million, with a total number of 4.43 million deaths, of which 1.43 million were new cases of cervical, ovarian, and endometrial cancers [72]. These data show the severe threat that common gynecological malignant tumors pose to women’s life and health. Thus, further comprehensive research on the pathogenesis, diagnostic markers, and therapeutic targets of gynecological malignant cancers is critical. The role of tsRNAs in gynecological malignant cancer has gradually been unveiled. Our review of previous works showed that recent research on the relationships between tsRNA and gynecological malignancies has been focused only on ovarian and cervical cancers, whereas no report is available on endometrial cancer. Most currently employed research approaches are associated with detecting and establishing differentially expressed tsRNAs through RNA sequencing and qRT-PCR verification. Then, further cell function experiments and mechanism analysis, or receiver operating characteristic (ROC) curve drawing, are implemented to determine the function and value of tsRNA as a diagnostic marker.

An earlier study was conducted to identify high-grade serous cystadenocarcinoma of the ovary and related tsRNAs and their function and mechanism of action in the SK-OV-3 ovarian cancer cell line [73]. The authors used small RNA sequencing and found that a total number of 27 tsRNAs were differentially expressed between patients with high-grade ovarian serous cystadenocarcinoma and healthy controls. The functions of these tsRNAs involved protein phosphorylation modification, transcription, affecting tumor cell migration and the MAPK and Wnt signaling pathways. Another investigation was performed to evaluate the diagnostic value of circulating tsRNAs in ovarian cancer by assessments of small RNA transcriptome sequencing data from the serum of patients with epithelial ovarian cancer, borderline and benign ovarian tumors collected from public databases, using healthy people as a control [74]. These researchers found a total number of 29 863 tsRNA, of which only four tsRNAs derived from tRNA-Gly were differentially expressed between ovarian cancer patients and healthy controls, which were consecutively named ts1–4. By ROC curve analysis, the area of the curve for the four tsRNA in the diagnosis of ovarian tumors alone was 0.836–0.948, of which ts-3 had an optimal performance. Nevertheless, further research is needed to determine the value of ts-3 as a diagnostic marker for ovarian cancer.

AlkB homolog 3(ALKBH3) is a tRNA methylase. Studies have shown that the increase in tsRNA expression depends on the upregulation of the expression of tRNA demethylase ALKBH3 in cells [75]. Sequencing of the tsRNA in HeLa cells stably expressing ALKBH3 and control cells was applied in a study to explore the function of ALKBH3 in a cervical cancer HeLa cell line. The scientists found that the overexpression of ALKBH3 enhanced the production of tsRNA in HeLa cells, with the prevalence of 5′tRF-GlyGCC. Cell function experiments revealed that 5′-tRF-GlyGCC enhanced the proliferation of wild-type HeLa cells and weakened the inhibitory effect of ALKBH3−/− cell proliferation. In addition, 5′-tRF-GlyGCC promoted the synthesis of proteins in HeLa cells. This evidence indicates that 5-‘tRF-GlyGCC is involved in the progression of ALKBH3-mediated cervical cancer [76].

## Summary and prospects

Research on tsRNA has now attracted significant research interest, establishing diverse their types and abundance of functions. According to their corresponding positions in the parental tRNA transcripts, they are classified into tRF-1s, tRF-3s, tRF-5s, tiRNA, and tRF-2s/i-tRFs [3]. Each particular type of tsRNA has a specific structure and production method. Their functions are various, including gene expression regulation, protein translation inhibition, anti-apoptotic effects, and participation in epigenetic regulation, initial virus reverse transcription, promote virus replication and cell-to-cell communication. In addition, the function of tsRNA also involves transgenerational inheritance [77]. Their role in malignant tumors has also been gradually revealed, which is critically important. tsRNAs are expected to be used as a marker for the diagnosis, progression, and prognosis of malignant tumors, and as a target for treatment, with broad application prospects. However, the exact underlying mechanisms of action and the corresponding functions of each tsRNA type have not been fully elucidated. Therefore, further research is needed to elucidate them. Meanwhile, with the development of high-throughput sequencing technology and the emergence of even more advanced research methods, increasingly more tsRNA will be discovered, with a high diversity of types and functions, which would extend their potential beneficial applications.

## Data Availability

The data used to support the findings of this study are available from the corresponding author upon request.
